# Ferroptosis regulators related scoring system by Gaussian finite mixture model to predict prognosis and immunotherapy efficacy in nasopharyngeal carcinoma

**DOI:** 10.3389/fgene.2022.975190

**Published:** 2022-09-02

**Authors:** Zijian Liu, Jinlan He, Xiaolin Hu

**Affiliations:** ^1^ Department of Head and Neck Oncology, Cancer Center, West China Hospital, Sichuan University, Chengdu, China; ^2^ West China School of Nursing, West China Hospital, Sichuan University, Chengdu, China

**Keywords:** ferroptosis, nasopharyngeal carcinoma, tumor microenvironment, Gaussian finite mixture model, prognosis, immunotherapy

## Abstract

The role of ferroptosis in tumor progression and metastasis has been demonstrated. Nonetheless, potential biological function of ferroptosis regulatory pattern in nasopharyngeal carcinoma (NPC) remains unknown. Ferroptosis regulatory patterns of nasopharyngeal carcinoma samples were evaluated based on 113 ferroptosis regulators and three distinct ferroptosis subtypes were determined by unsupervised clustering. The ferroptosis score (FEP score) was identified to quantify ferroptosis patterns within individual tumors by Gaussian finite mixture model and systematically correlated with representative tumor characteristics. Subtype 1 and subtype 3 were consistent with immune activated phenotype, while subtype 2 was consistent with immune suppressed phenotype. High ferroptosis score, characterized by immune activation and suppression of mRNA based stemness index (mRNAsi) and Epstein-Barr virus (EBV) genes, indicated an immune activated tumor microenvironment (TME) phenotype, with better progression free survival (PFS) and lower risk of recurrence and metastasis. Low ferroptosis score, characterized by activation of Wnt and NF-κB signaling pathways and lack of effective immune infiltration, indicated an immune suppressed tumor microenvironment phenotype and poorer survival. High ferroptosis score was also correlated to enhanced response to immunotherapy, and was confirmed to correlate with therapeutic advantages and clinical benefits in an anti-programmed cell death 1 ligand 1 (PD-L1) immunotherapy cohort. As ferroptosis played a crucial role in the tumor microenvironment’s diversity, assessing the ferroptosis pattern within individual tumor with ferroptosis score could enhance our understanding of tumor microenvironment infiltration characterization and help develop more effective immunotherapy.

## Introduction

Nasopharyngeal carcinoma (NPC), originating from the squamous epithelial cells of the nasopharyngeal mucosa, is a malignancy characterized by a distinct racial and geographical distribution which is highly prevalent in east and southeast Asia (Zhang et al., 2015; Huang S. J. et al., 2018; Chen Y. P. et al., 2019). NPC is etiologically associated with Epstein-Barr virus (EBV) infection (Kamran et al., 2015), and exhibits considerable immune cell infiltration in tumor microenvironment (TME) (Zhang et al., 2010), making immunotherapy a promising treatment for patients with NPC. However, although immunotherapy targeting the immune checkpoints have proven to be effective in multiple tumor types (Hargadon et al., 2018), the efficacy of immune checkpoint inhibitors (ICIs) is far from satisfying in NPC patients in early phase clinical trials (Hsu et al., 2017; Fang et al., 2018). Given the significant heterogeneity in the EBV status and TME characteristics (Huang S. C. M. et al., 2018) in NPC, it is worth studying whether these differences cause distinct immunotherapy responses. Moreover, biological factors regulating the TME remains to be elucidated.

Ferroptosis is a recently recognized iron-dependent programmed cell death involving lethal iron-catalyzed lipid damage, and is regulated by numerous genes classified as suppressors of ferroptosis (SOFs), drivers of ferroptosis (DOFs), and markers of ferroptosis (MOFs) (Dixon et al., 2012; Hassannia et al., 2019). Dysfunctional ferroptosis is involved in the development of numerous human diseases including carcinogenesis (Stockwell et al., 2017). Owing to its key role in tumor inhibition (Yang et al., 2016; Yang and Stockwell, 2016), ferroptosis has become a hopeful therapeutic target in cancer treatment (Yamaguchi et al., 2013; Ooko et al., 2015). Recent studies reported the close interaction between ferroptosis and immune system, and highlighted ferroptosis as a promising approach for immunotherapy. For example, immunotherapy-activated CD8^+^ T cells could enhance ferroptosis and further improve the efficacy of immunotherapy by downregulating two subunits of the glutamate-cystine antiporter system x_c_^−^ (Wang et al., 2019), suggesting that the immune system might function partly through ferroptosis (Stockwell and Jiang, 2019). Moreover, the release of immunomodulatory signals such as oxidized lipid mediators might influence antitumor immunity, or a small part of cells in the tumor bulk undergoing ferroptosis might lead to immune evasion (Friedmann Angeli et al., 2019). Understanding the ferroptosis patterns and its effect on TME as well as immune response would better help guide the application of immunotherapy.

Investigations on ferroptosis are limited in NPC. It has been reported that some drugs triggering ferroptosis could attenuate the progression and stemness of NPC cells (Li et al., 2020; He et al., 2021; Huang et al., 2021; Xu et al., 2021). However, these researches only focused on the function of a single ferroptosis-related molecule or pathway, nor did they reveal the effect of ferroptosis on TME and immune response. On the other hand, recent studies revealed that ferroptosis related gene signatures are related with both the prognosis and immune cell infiltration levels in hepatocellular carcinoma (Liu Y. et al., 2020; Du and Zhang, 2020; Tang et al., 2020). Therefore, the present study aims to determine the ferroptosis regulatory patterns and related biological characterizations in NPC. First, the genomic information as well as clinical traits of patients with NPC from public database were integrated to synthetically assess the ferroptosis regulatory patterns and their corresponding characteristics of TME. Then, the functional network between ferroptosis regulators and ferroptosis related genes together with underlying regulatory modifier genes was conducted. Furthermore, a ferroptosis score (FEP score) was developed and validated to predict potential responses to immunotherapy.

## Materials and methods

### Dataset source and data preprocessing

The raw gene expression data were obtained from Gene Expression Omnibus (GEO) database (https://www.ncbi.nlm.nih.gov/geo/)Included datasets were listed in Supplementary Table S1, among which five NPC datasets (GSE12452, GSE34573, GSE53819, GSE64634 and GSE68799) were used for further analysis. Microarray data were obtained as the raw “CEL” files from GEO before normalization and analysis, while high throughput sequencing data were directly downloaded. Data on somatic mutation as well as copy number variation were downloaded directly from supplementary materials from a genomic analysis of NPC (Zhang et al., 2017). A cohort of patients with advanced urothelial cancer treated with atezolizumab, an anti-programmed cell death 1 ligand 1 (PD-L1) antibody (IMvigor210 cohort) was used as the immunotherapeutic cohort for validation (Mariathasan et al., 2018), and data on gene expression and clinical annotations was obtained according to the Creative Commons 3.0 License from http://research-pub.Gene.com/imvigor210corebiologies. Gene expression values in the form of fragments per kilobase per million (FPKM) and clinical data of pan-cancer including17 cancer types in the Cancer Genome Atlas (TCGA) database were downloaded from University of California Santa Cruz (UCSC) XENA database (https://xenabrowser.net/datapages/) (Goldman et al., 2020).

### Differential gene expression analysis and gene ontology (GO) analysis

Differential gene expression analysis between different defined groups was conducted using the empirical Bayesian approach of “limma” R package and the significance criteria was defined as adjusted *p* value <0.05 and Log2 fold-change (log_2_FC) > 1. The differentially expressed mRNAs were shown in heatmap and volcano plot in R using “pheatmap” and “ggplot2” packages. GO and Kyoto Encyclopedia of Genes and Genomes (KEGG) analyses were conducted using the clusterProfiler package of R software.

### Unsupervised clustering for ferroptosis regulators

A total of 113 ferroptosis regulators with validated confidence level in Homo sapiens experiment were extracted from an online website FerrDb (http://www.zhounan.org/ferrdb/), including 49 SOFs, 61 DOFs and 3 MOFs, and the specific information of these genes were shown in Supplementary Table S2. Unsupervised clustering analysis was performed according to the expression of the 113 ferroptosis regulators and used to identify distinct ferroptosis regulatory patterns and classify patients. The number and stability of clusters were determined with the consensus clustering algorithm. To guarantee the stability of classification, ConsensuClusterPlus package was applied and 1,000 repetitions were conducted (Wilkerson and Hayes, 2010).

Implementation of single sample gene set enrichment analysis (ssGSEA).

The gene set enrichment analysis (GSEA) program was used to calculate the absolute enrichment scores of validated gene signatures of a single sample. In brief, the enrichment score of both biological process and infiltration immune cells were quantified by ssGSEA in R package “gene set variation analysis (GSVA)”, a non-parametric and unsupervised method for estimating variation of gene set enrichment of a single sample (Hänzelmann et al., 2013). Both the gene set of “c5. all.v6.2. Symbols” downloaded from the Molecular Signatures Database (MSigDB) and another published gene set storing genes associated with some biological processes (Mariathasan et al., 2018) were utilized to run GSVA for underlying biological function prediction. Additionally, the relative abundance of infiltration of each kind of immune cell in the TME of NPC was calculated using ssGSEA algorithm with a set of immune cell markers published in articles, containing 23 types of immune cells (Charoentong et al., 2017). To roughly assess EBV gene expression, genes notably correlated with EBV genes (Pearson coefficient >0.3) (Zhang et al., 2017) instead of EBV genes were extracted for ssGSEA analysis because the profile of EBV gene expression was not uploaded. The above gene sets and immune cell markers were shown in Supplementary Table S3–5.

### Calculation of ferroptosis index and ferroptosis score

To represent the ferroptosis level, a ferroptosis index (FPI) was established based on the expression data of genes in ferroptosis with positive components including LPCAT3, ACSL4, NCOA4, ALOX15, GPX4, SLC3A2, SLC7A11, NFE2L2, NOX1, NOX3, NOX4, NOX5 and negative components including FDFT1, HMGCR, COQ10A, COQ10B. The enrichment score (ES) of gene set positively or negatively regulating ferroptosis was calculated using ssGSEA, and the FPI was calculated as follows:

FPI = ES (positive)—ES (negative) (Liu Z. et al., 2020).

To quantify the ferroptosis regulatory patterns, FEP score was calculated using principal component analysis (PCA). Principal component 1 (PC1) and principal component 2 (PC2) of each sample were calculated using the expression matrix of genes with prognostic significance (gene i) (Zhang et al., 2020). The FEP score was calculated as follows:

$$\text{FEP}\;\text{score}=\sum \left(\text{PC}1_{\text{i}}\;+\text{ PC}2_{\text{i}}\right)$$

### Prediction of immunotherapy response for patients

Tumor Immune Dysfunction and Exclusion (TIDE) database (http://tide.dfci.harvard.edu/) was used to predict patients’ response to immunotherapy (Jiang et al., 2018). The calculated TIDE value was used to assess the probability of immunotherapy response with a cutoff value defaulted as 0. As the input data needs to be normalized and melanoma as well as non-small cell lung cancer (NSCLC) were the suggested tumor types, the results could only be auxiliary.

### Calculation of gene expression based stemness index (mRNAsi) for patients

To evaluate the stemness of cancer cells, the mRNAsi was calculated with a one-class logistic regression algorithm in each NPC sample (Malta et al., 2018). The mRNA expression-based signature consisted a gene expression profile including11 774 genes, and the workflow to generate the stemness index was from established database (https://bioinformaticsfmrp.github.io/PanCanStem_Web/). We applied the mRNAsi to score the NPC samples using Spearman correlation and the stemness index was mapped to the (0,1) range afterward via a linear transformation as reported (Malta et al., 2018).

Construction of the network among ferroptosis and N6-methyladenosine (m6A) modification genes.

A total of 26 RNA m6A regulators were obtained from articles which identified different m6A modification patterns in NPC (Li et al., 2019; Lu et al., 2020; Zhang et al., 2020), including 10 writers (KIAA1429, WTAP, RBM15, RBM15B, ZC3H13, METTL3, METTL5, METTL14, METTL16, CBLL1), 14 readers (ELAVL1, FMR1, HNRNPA2B1, HNRNPC, IGF2BP1, IGF2BP2, IGF2BP3, LRPPRC, RBMX, YTHDC1, YTHDC2, YTHDF1, YTHDF2, YTHDF3) and two erasers (ALKBH5, FTO). Protein-protein interaction network was constructed in STRING database with median confidence and visualized in Cytoscape software.

### Gaussian mixture and logistic regression model construction

Clustering was conducted based on the Gaussian finite mixture model (GFMM) (Hong H. C. et al., 2020). The ferroptosis regulators clusters were classified by GFMM. Logistic regression analysis was then used to construct combined models to predict FEP score groups. Furthermore, a nomogram made up of the seven ferroptosis regulators was built through the R package “rms” to predict the progression free survival (PFS) probability.

### Statistical analysis

Correlation coefficients and *p* values among groups were obtained using Spearman correlation analysis. Comparisons among three or more groups were conducted using Kruskal–Wallis tests, and comparisons between two groups were performed using Wilcoxon tests. The “surv-cutpoint” function was utilized to decide the optimal separation cutoff value in survival analysis using the “survminer” R package. Survival curves were generated using the Kaplan-Meier method and compared between groups via the log-rank tests. Least Absolute Shrinkage and Selector Operation (LASSO) algorithm was used to select candidate ferroptosis genes and ferroptosis related genes. Waterfall function of “maftools” package was used to visualize the mutation landscape of samples in patients with NPC. All data processing was performed in R 4.0.3 software, with two-side *p* values < 0.05 considered statistically significant.

## Results

### Ferroptosis regulatory patterns mediated by ferroptosis regulators

**FIGURE 1:**
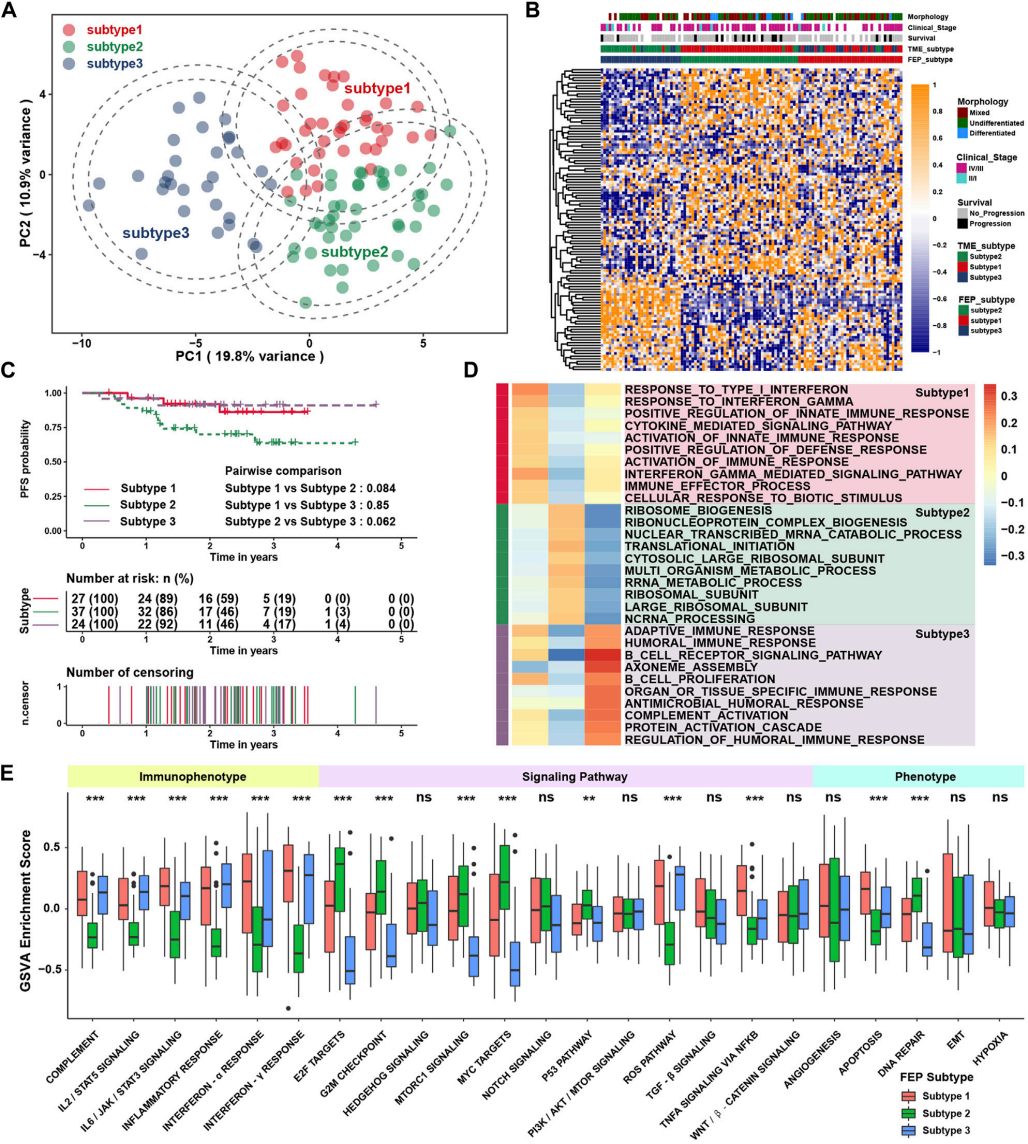
Identification of ferroptosis regulatory patterns and biological function analysis. **(A)** The transcriptome profiles of ferroptosis subtypes using principal component analysis. **(B)** Heatmap of ferroptosis regulators among ferroptosis subtypes. Morphology, clinical stage, survival, and TME subtype were used as patient annotations. **(C)** Survival analysis among ferroptosis subtypes in GSE102349. **(D)** GSEA revealed distinct enriched gene sets among subtypes. Rows were defined by gene sets, and columns by consensus scores for each subtype. **(E)** The GSVA enrichment score in pathways. Each box spans the interquartile range with the upper and lower end of the boxes representing the 25th and 75th percentile values. The horizontal lines in the boxes represented the median values. The black dots showed outliers. The asterisks represented *p* values (**p < 0.01; ***p < 0.001) and ns represented no significance.

In total, 113 ferroptosis regulators (49 SOFs, 61 DOFs and 3 MOFs) were involved in this study, and the main workflow was shown in Supplementary Figure S1. To explore the regulatory patterns of the ferroptosis regulators in NPC, patients were classified with qualitatively different ferroptosis regulatory patterns based on the expression of 113 ferroptosis regulators using the R package of ConsensusClusterPlus, and three distinct patterns were identified using unsupervised clustering, including 39, 44 and 30 cases in subtype 1, subtype 2 and subtype 3, respectively (Supplementary Figure S2). The expression of ferroptosis regulators were significantly different among three subtypes, as shown in the PCA and heatmap (Figures 1A,B). The survival curves of three ferroptosis subtypes were distinctive, and the prognosis of patients in subtype 2 seemed to be worst, although without statistical differences (Figure 1C). To explore the underlying mechanism of ferroptosis subtypes, gene set enrichment analysis (GSEA) with distinct enriched gene sets was conducted among above subtypes (Figure 1D). The immune related pathways were highly activated in subtype 1 and 3, while these pathways were not enriched in subtype 2, which might be the reason for the poor prognosis in subtype 2. To validate the biological function variation among subtypes, GSVA was performed. As shown in Figure 1E, ferroptosis subtype 1 and subtype 3 were markedly enriched in immunophenotype including interferon-α/γ, IL-2/STAT5, complement and apoptosis pathways, while ferroptosis subtype 2 presented enrichment in pathways associated with E2F, G2M, MYC and DNA repair. The above results indicated that ferroptosis regulatory patterns might be associated with regulation of immune and cell proliferation related phenotypes.

### Immune cell infiltration characteristics in distinct ferroptosis subtypes

**FIGURE 2:**
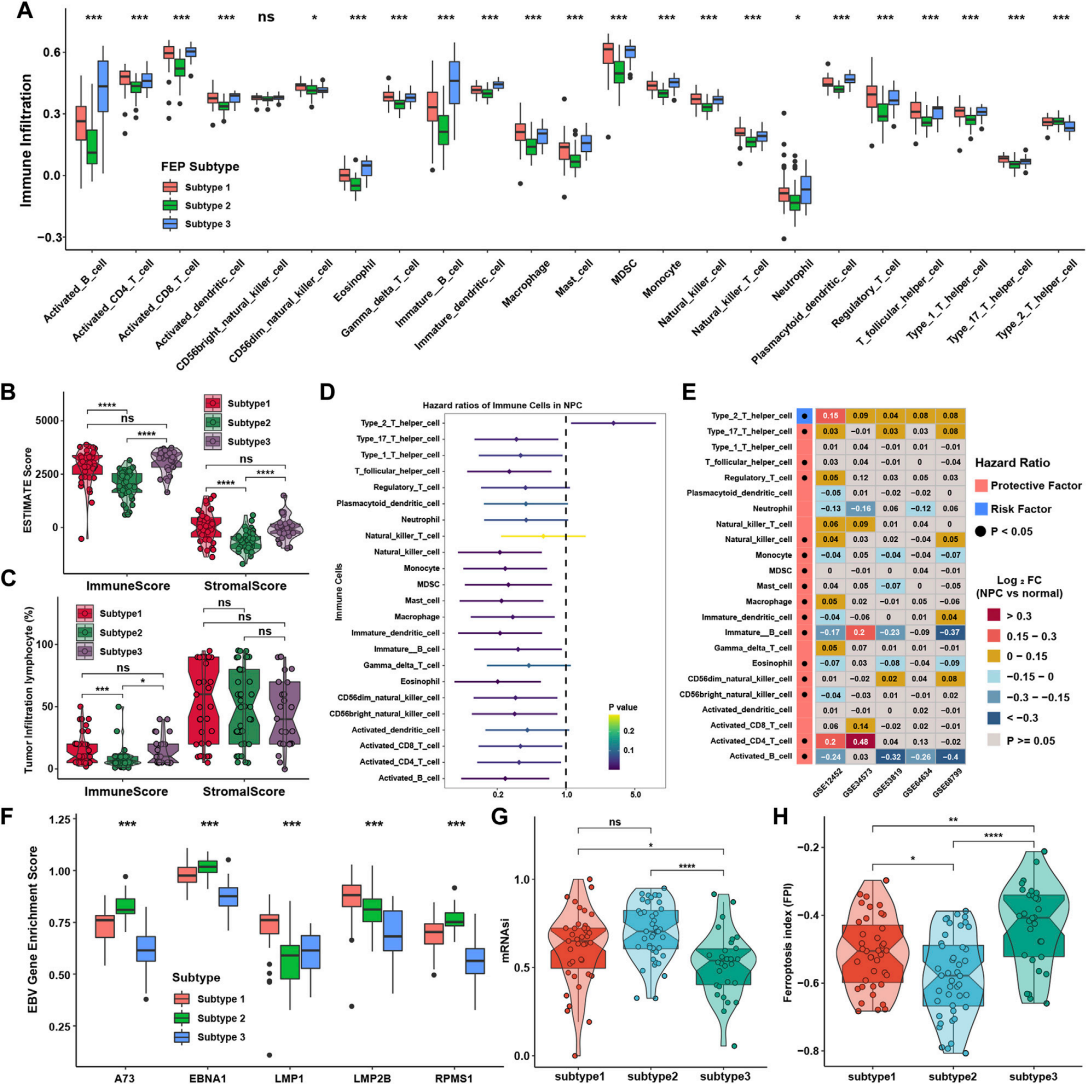
Immune cell infiltration characteristics in distinct ferroptosis subtypes. **(A)** Immune cell infiltration levels in TME among ferroptosis subtypes. **(B)** Immune and stromal scores via ESTIMATE among ferroptosis subtypes. **(C)** Intratumor and stromal tumor infiltration lymphocytes provided in GSE102349 among ferroptosis subtypes. **(D)** The prognostic analyses for tumor-infiltrating immune cells in TME in GSE102349 cohort, and hazard ratio >1 and <1 represented risk and protective factor for survival, respectively. **(E)** Differential expression analysis of TME infiltration in five independent datasets. Black dots in the left column represented *p* < 0.05. **(F)** The GSVA enrichment score of EBV genes among ferroptosis subtypes. **(G)** The mRNAsi among ferroptosis subtypes. **(H)** FPI among ferroptosis subtypes. The asterisks represented *p* values (*p < 0.05; **p < 0.01; ***p < 0.001; ****p < 0.0001) and ns represented no significance.

SsGSEA was applied to calculate relative expression level of immune cells with specific immune cell signatures, and the results showed that subtype 1 and subtype 3 were notably rich in immune cells including activated CD4 + and CD8 + T cells, activated dendritic cells (DCs) and B cells, except type 2 T helper cells, which was highly enriched in subtype 2 (Figure 2A). These results reflected there were both activated immune pathways and enriched immune cells in ferroptosis subtype1 and subtype 3. With Estimation of Stromal and Immune cells in malignant tumors using Expression data (ESTIMATE) method, it is found that stromal and immune scores were higher in tumor samples in subtype 1 and subtype 3, which was consistent with TME infiltration analysis (Figure 2B). The percentage of tumor infiltration lymphocytes was also compared as defined in previous study (Zhang et al., 2017). Although there was no significant difference in the average percentage of stromal lymphocytes among three subtypes, the average percentage of intra-tumoral lymphocytes were significantly higher in subtype 1 and subtype 3 (Figure 2C). To further investigate whether the immune cell infiltration would affect PFS, survival analysis was performed and found that only the infiltration level of type 2 T helper cell was significantly a risk factor for PFS, while other types of immune cells were protective factors (Figure 2D). Therefore, we analyzed the levels of immune infiltration between NPC and normal samples in five NPC datasets to investigate the dominant cells in the TME of NPC. Surprisingly, the infiltration levels of type 2 and type 17 T helper cells were significantly higher in NPC samples, while activated and immature B cells, the main target cells of EBV in the initiation of NPC, were consistently lower in NPC samples (Figure 2E). This promoted the analysis of relative expression of EBV genes using ssGSEA in GES102349 dataset. Interestingly, there were indeed some differences in the expression of EBV genes among ferroptosis subtypes, although the differences were somewhat inconsistent among these genes (Figure 2F). The enrichment of pathways highly associated with tumor initiation and metastasis in subtype 2 sparked the interest in studying the mRNA based stemness index (mRNAsi), and the result showed that the mRNAsi was higher in subtype 2 than in the other two subtypes, indicating that subtype 2 possessed higher capability of invasion and metastasis (Figure 2G). Ferroptosis index (FPI), a calculated method published before (Liu Z. et al., 2020), was used to assess ferroptosis levels among ferroptosis subtypes, and found that the FPI was significantly higher in subtype 1 and subtype 3 (Figure 2H). These results displayed that ferroptosis might be associated with immune cell infiltration, EBV infection, and metastasis phenotypes.

### Generation of ferroptosis score and functional annotation

**FIGURE 3:**
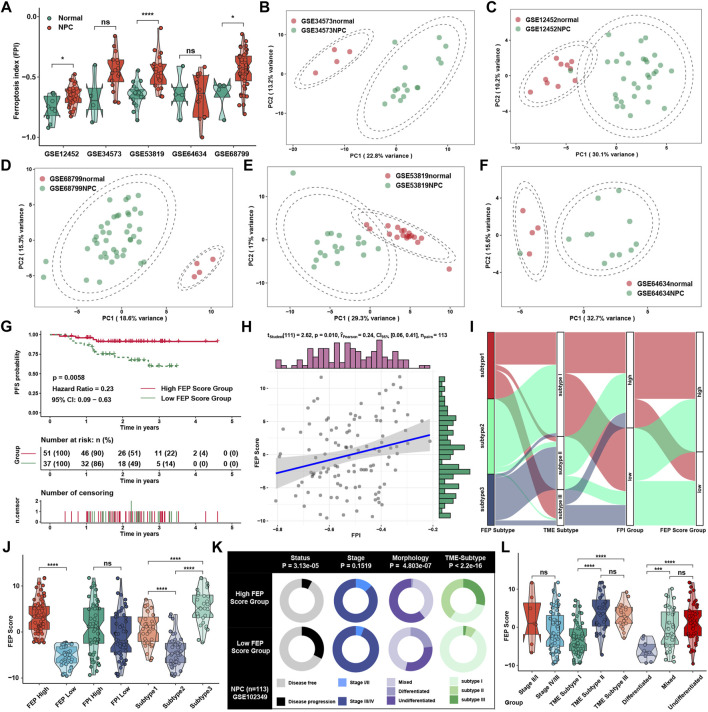
Generation of FEP score and functional annotation. **(A)** Differences of FPI in NPC samples in comparison with normal tissue in five independent NPC datasets in GEO database, including GSE12452, GSE34573, GSE53819, GSE64634 and GSE68799. **(B–F)** Principal component analysis for the ferroptosis regulators in five independent datasets, showing a remarkable difference in mRNA expression between normal tissues and NPC. **(G)** Survival analysis for patients stratified by FEP score in GSE102349. **(H)** Correlation analysis between FPI and FEP score. **(I)** Alluvial diagram visualizing the changes of ferroptosis subtypes, TME subtypes, FPI and FEP score. **(J)** FEP score among different ferroptosis subtypes, FPI and FEP score groups. **(K)** Clinical characterization in high and low FEP score groups. The chi-square test was used to calculate statistical differences. **(L)** FEP score among different clinical stages, TME subtypes and morphologies. The asterisks represented *p* values (*p < 0.05; ***p < 0.001; ****p < 0.0001) and ns represented no significance.

To investigate the ferroptosis level between NPC and normal tissue, we found that FPI was significantly higher in NPC than normal tissue (Figure 3A), which was consistent with the results in pan-cancer in previous study (Liu Z. et al., 2020), suggesting that ferroptosis might be critical in the progression of NPC. Principal component analysis revealed that the expression of ferroptosis regulators could well reflect the differences between NPC and normal samples (Figures 3B–F). Considering the individual heterogeneity and complexity in the regulatory patterns of ferroptosis, FEP score, a set of scoring system, was generated with ferroptosis regulators to quantify the ferroptosis regulatory level in individual patients with NPC. Patients were classified into high or low FEP score group by “survminer” package, and those with high FEP score demonstrated a prominent survival benefit (Figure 3G). Correlation analysis showed that FEP score might retain part of characteristics of FPI, and FEP score was positively correlated with FPI (Figure 3H), which might mean that FEP score was a novel scoring system different from FPI to assess the biological function of ferroptosis. The alluvial diagram was applied to visualize the attribute changes of individual patients (Figure 3I), and indicated that FEP score might be the best way to present ferroptosis regulatory patterns at the individual level. Indeed, FEP score could reflect the grouping of ferroptosis subtypes and FEP score groups well, while no statistical difference in FEP score was shown between FPI groups (Figure 3J). To better illustrate the characteristics of FEP score, the correlations between clinical traits and the FEP score were examined, and the result showed that high FEP score was significantly correlated with disease free status, mixed and undifferentiated morphology, and immune activated TME subtypes (Figure 3K). In terms of clinical characteristics, FEP score was higher in TME subtype II and III as well as mixed and undifferentiated morphology (Figure 3L), which were groups with better prognosis and lower progression risk.

### Characteristics and biological function of ferroptosis score in nasopharyngeal carcinoma

**FIGURE 4:**
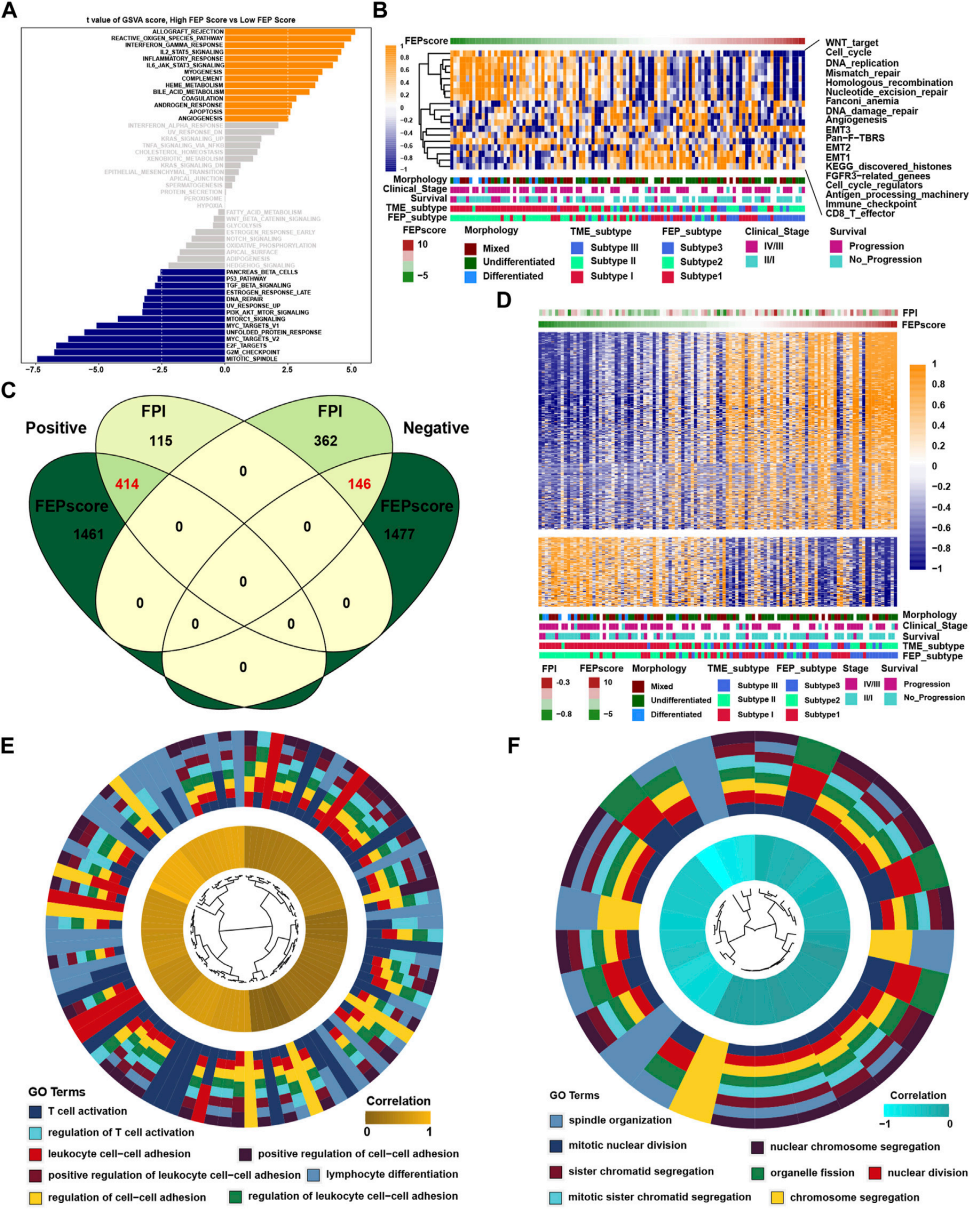
Characteristics and biological function of FEP score in NPC. **(A)** GSVA score for pathways in low and high FEP score groups. The orange and blue columns represented pathways enriched in high and low FEP score group, respectively. **(B)** Correlations between FEP score and known gene signatures in NPC using Spearman analysis. Positive and negative correlations were marked with orange and blue, respectively. **(C)** Venn plot of candidate ferroptosis related genes and the red fonts represented the intersection genes correlated with FPI and FEP score simultaneously. **(D)** Heatmap of candidate ferroptosis related genes among ferroptosis subtypes. Morphology, clinical stage, survival, and TME subtypes were adopted as annotations. **(E–F)** GO analysis of candidate ferroptosis genes positively or negatively correlated with FPI and FEP score.

GSVA showed that in immune related pathways such as IL-2/STAT5, IL-6/JAK/STAT3 and interferon response pathways were enriched in high FEP score group, while E2F, G2M and MYC related pathways were enriched in low FEP score group (Figure 4A). To further verify the above underlying biological function, previously known pathway signatures was used to clarify the correlation between FEP score and the enrichment score of specific pathways (Figure 4B). The results indicated that FEP score positively correlated with immune related pathways and negatively correlated with Wnt, cell cycle, DNA damage repair and homologous recombination pathways. Correlation analysis was conducted between FPI, FEP score and mRNA expression in GSE102349 to explore ferroptosis related genes. A total of 414 genes were found to be positively correlated with FPI and FEP score and 146 genes were negatively correlated with FPI and FEP score simultaneously (Figures 4C,D). GO analysis showed that the positively correlated genes were enriched in T cell activation and cell-cell adhesion biological function and the negatively correlated genes were enriched in mitotic nuclear division and chromosome segregation (Figures 4E,F).

### Role of ferroptosis score in immunotherapy for nasopharyngeal carcinoma

**FIGURE 5:**
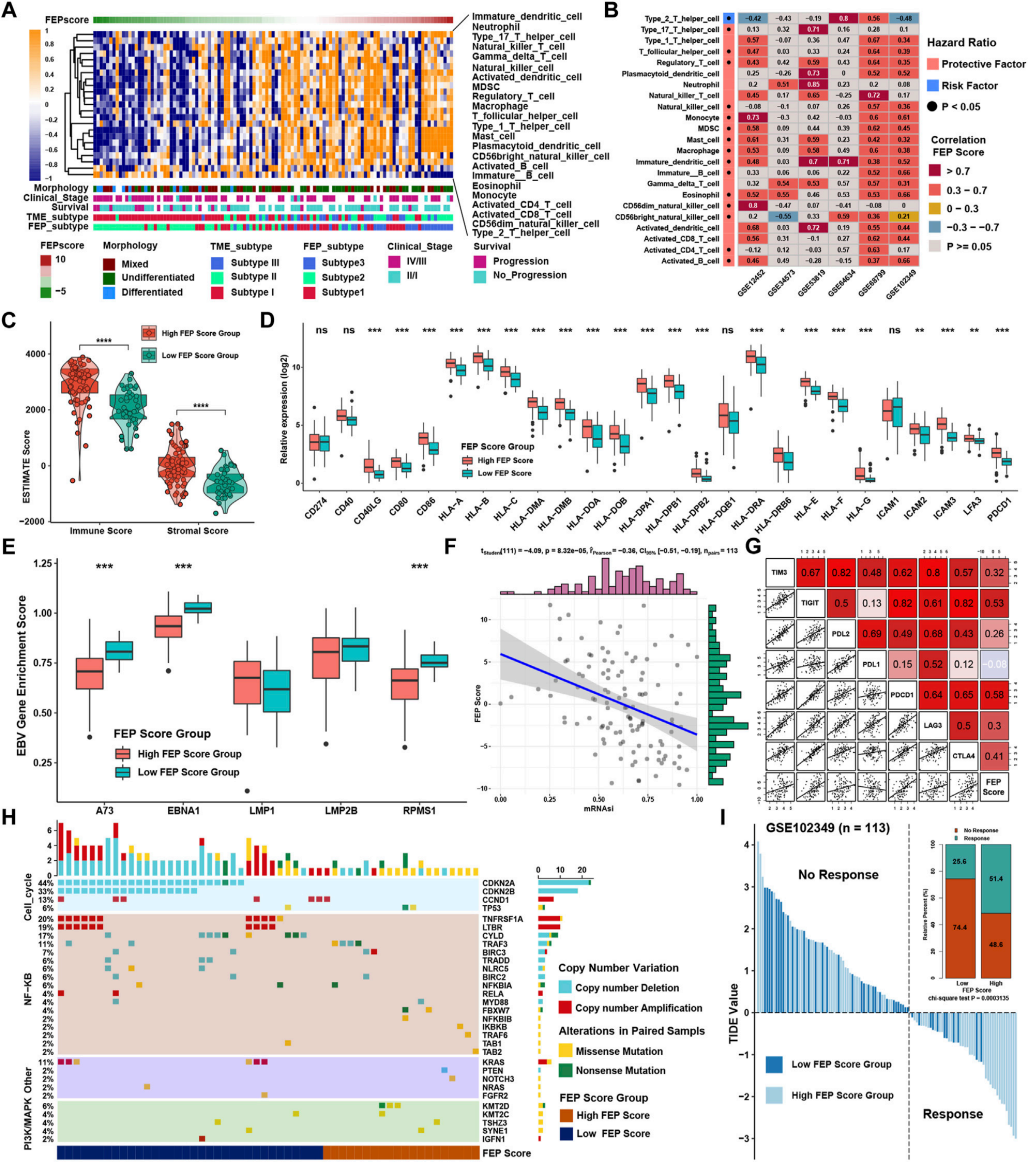
Role of FEP score in immunotherapy for NPC. **(A)** Correlations between FEP score and the immune cell infiltrations in NPC using Spearman analysis. Positive and negative correlations were marked with orange and blue, respectively. **(B)** Correlations between FEP score and TME infiltration in six independent NPC datasets using Spearman analysis. **(C)** ESTIMATE ps. **(D)** The relative expression of costimulatory, MHC, and adhesion molecules in different FEP score groups. **(E)** EBV gene enrichment score by GSVA in different FEP score groups. **(F)** Correlation analysis between FEP score and mRNAsi using Spearman analysis. **(G)** Correlation analysis between FEP score and immune checkpoint inhibitors in GSE102349 using Spearman analysis. **(H)** Somatic copy number variations and mutations in the paired NPC cohort in GSE102349 were shown in different FEP score groups. **(I)** TIDE value of NPC samples in GSE102349 in different FEP score groups. The chi-square test was used to calculate statistical differences. The asterisks represented *p* values (*p < 0.05; **p < 0.01; ***p < 0.001) and ns represented no significance.

**FIGURE 6:**
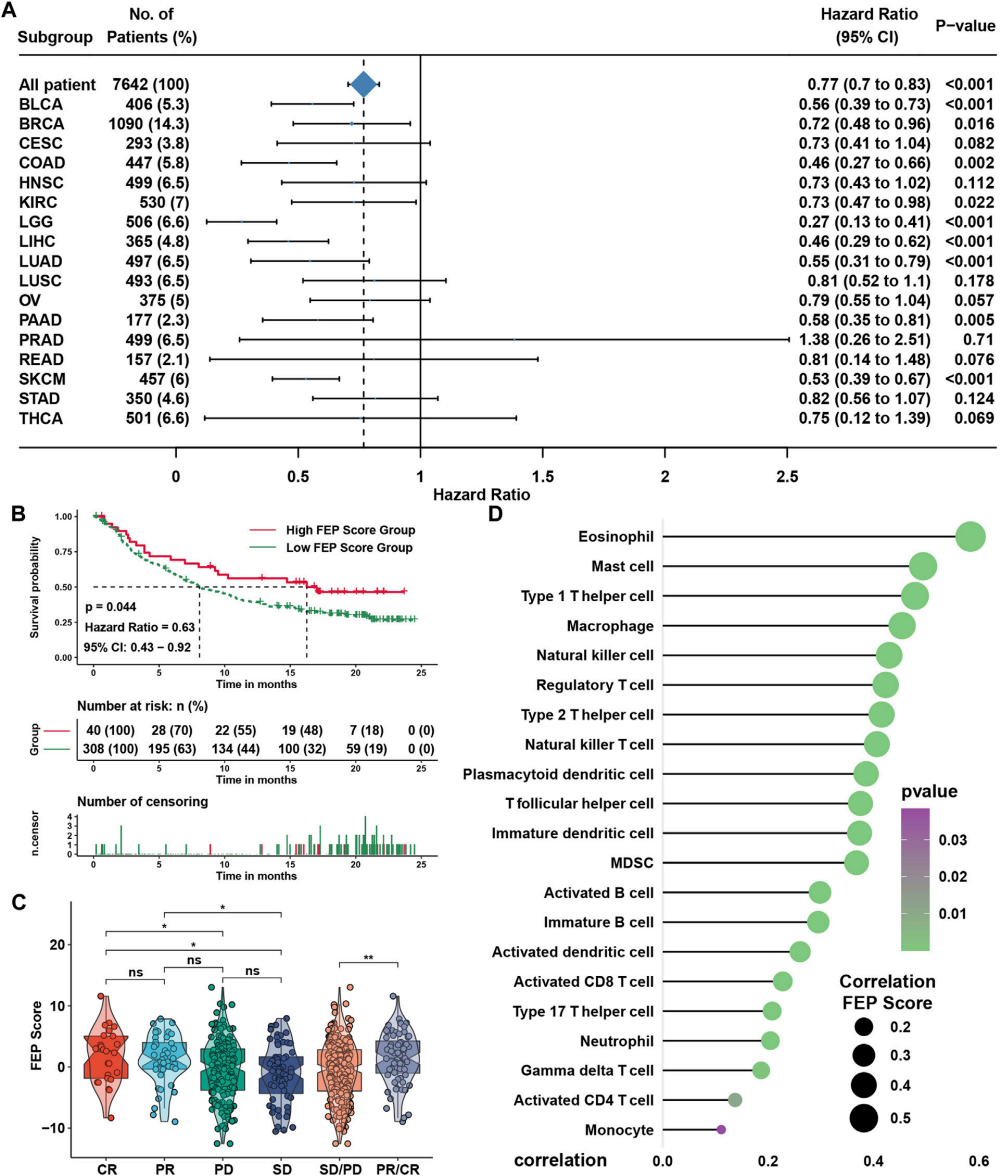
FEP regulatory patterns in pan-cancer and the role in immunotherapy. **(A)** Subgroup analyses evaluating prognostic value of FEP score in various types of cancer from TCGA datasets. HR > 1.0 indicated high FEP score as a protective prognostic factor. **(B)** Survival analysis for FEP score in IMvigor210 cohort. **(C)** Distribution of FEP score in groups with distinct clinical responses to anti-PD-L1 treatment. **(D)** Correlation analysis between FEP score and infiltration levels of immune cell in IMvigor210 cohort. The asterisks represented the *p* values (*p < 0.05; **p < 0.01).

SsGSEA showed that the infiltration levels of most immune cells were highly positively associated with FEP score, except type 2 T helper cell (Figure 5A). Considering the potential function of FEP score in immune response, FEP scores were calculated, and the correlation between FEP score and infiltration levels of immune cells were enriched by ssGSEA in six NPC datasets (Figure 5B). The results revealed that FEP score was positively correlated with most immune cells. Further verification using ESTIMATE algorithm also found that both immune and stromal scores were higher in high FEP score group (Figure 5C). Previous study showed that the activation of dendritic cells (DCs), the key antigen-presenting cells responsible for activation of naive T cells, depended on the high expression of costimulatory molecules, major histocompatibility complex (MHC) molecules, and adhesion molecules (Qian and Cao, 2018). All the three categories of molecules were mostly highly expressed in high FEP score group (Figure 5D). Interestingly, the expression of EBV genes such as A73, EBNA1 and PRMS1 were significantly higher in low FEP score group (Figure 5E), suggesting a possible correlation between worse prognosis as well as lower immune cell infiltration level and EBV infection. Correlation analysis further confirmed had a strong negative correlation between FEP score with mRNAsi, which could well reflect the stemness of cancer cells (Figure 5F). Furthermore, the correlation between FEP score and expression level of immunological checkpoint molecules were tested (Figure 5G). Studies have reported that activation of NF-κB pathway and cell cycle inhibitors played an important role in NPC (Zheng et al., 2016; Li et al., 2017; Wang et al., 2017) and deletion of several NF-κB and cell cycle inhibitors were found such as CDKN2A, CDKN2B, CYLD and TRAF3 (Zhang et al., 2017). Reanalyzing the copy number and mutations data in GSE102349, it was confirmed that the deletion frequencies of NF-κB and cell cycle inhibitors were higher in low FEP score group, but the mutations were not common in cell cycle, NF-κB or PI3K/MAPK pathways (Figure 5H). As expected, FEP score positively correlated with checkpoint molecules, suggesting a possibility of better response to immunotherapy in high FEP score group. Therefore, NPC patients were classified into response and no response groups with TIDE value to predict the immune response, and high FEP score group might have better response to immunotherapy (Figure 5I). Above evidences illustrated that high FEP score group with low probability of progression might be associated with more immune cell infiltration and better response to immunotherapy, while low FEP score group with high possibility of metastasis might possess more activated NF-κB pathway and higher mRNAsi. Considering the possible role of FEP score in predicting response to immunotherapy, whether the FEP score could predict patients’ response to ICIs was investigated in an immunotherapy cohort. Firstly, FEP score was proven a protective prognostic factor in all the 17 types of independent cancers in TCGA cohorts (Figure 6A). Thus, we further validated the predictive role of FEP score in response to ICIs in an anti-PD-L1 cohort (IMvigor210) in urinary carcinoma instead because of the lack of cohorts treated with immunotherapy in NPC. Survival analysis showed patients with high FEP score had better survival (Figure 6B). FEP score in complete response (CR) group was significantly higher than those in progressive disease (PD) or stable disease (SD) groups (Figure 6C). Correlation analysis indicated that FEP score also positively correlated with immune cell infiltration (Figure 6D).

### Identification of feature ferroptosis genes and construction of prediction model

**FIGURE 7:**
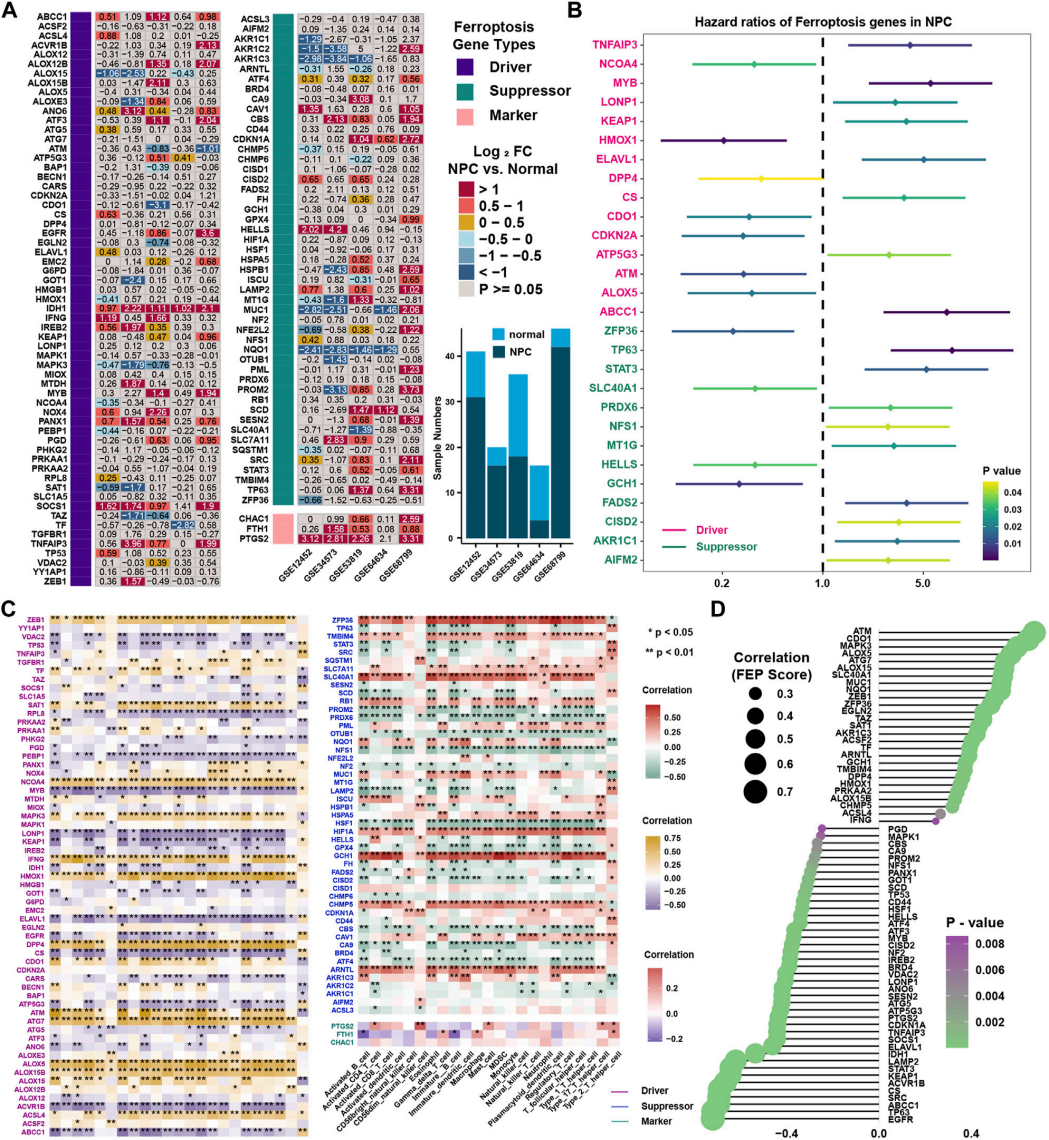
Identification of candidate ferroptosis genes. **(A)** Differentially expressed ferroptosis regulators in NPC samples in comparison with normal tissue in five independent NPC datasets including GSE12452, GSE34573, GSE53819, GSE64634 and GSE68799 in GEO database. **(B)** The prognostic analyses of 113 ferroptosis regulators in GSE102349 cohorts. Hazard ratio >1 and <1 represented risk factor and protective factor for survival, respectively. **(C)** Correlation analysis between ferroptosis regulators and tumor-infiltrating immune cells in TME using Spearman analysis in GSE102349. **(D)** Correlation analysis between FEP score and ferroptosis regulators.

**FIGURE 8:**
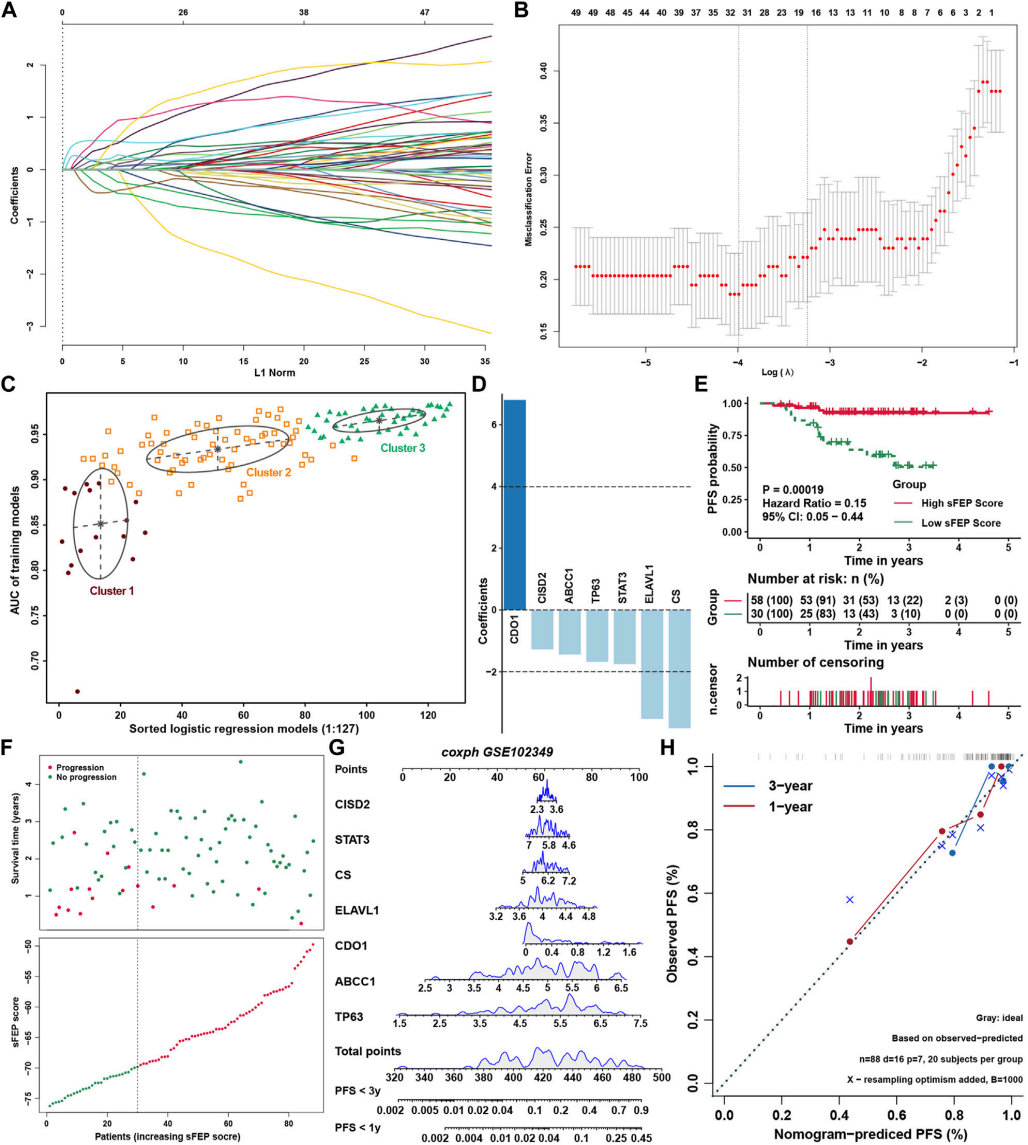
Construction of prediction model. **(A)** LASSO coefficient profiles of ferroptosis regulators. **(B)** Ten-time cross-validation for tuning parameter selection in the LASSO model for ferroptosis regulators. **(C)** The pattern of the logistic regression model correlated with the AUC scores identified by a Gaussian mixture. There are 3 clusters of 127 combinations. **(D)** The coefficients of seven genes involved in sFEP score signature. **(E)** Survival analysis for sFEP score in GSE102349. **(F)** The sFEP score distribution in the patients with NPC and the progression status of NPC from different groups of sFEP scores. **(G)** The nomogram to predict one-year and three-year PFS outcomes of NPC patients. **(H)** The calibration curve to evaluate the accuracy of the nomogram constructed based on sFEP signature.

To reveal the expression landscape of ferroptosis regulators between NPC and normal nasopharyngeal tissue, differentially distribution of mRNA expression of ferroptosis regulators was investigated by integrated bioinformatics analysis. Differentially expressed gene analysis was conducted in 5 GEO datasets (GSE12452, GSE34573, GSE53819, GSE64634 and GSE68799) between NPC and normal control samples, and the result showed that the expression of ferroptosis regulators stratified with the criteria log_2_FC > 1 and false discovery rate (FDR) < 0.05 were highly heterogenous in NPC (Figure 7A). Differentially expressed ferroptosis regulators were defined only if they have the same tendency of expression in at least three datasets. As a result, ABCC1, ANO6, IDH1, IREB2, PANX1, SOCS1, TNFAIP3, CBS, CDKN1A, LAMP2, SRC, FTH1 and PTGS2 were significantly upregulated, while ALOX15, MAPK3, AKR1C3, MUC1, NQO1 were significantly downregulated in NPC. Then, survival analysis was conducted in 88 NPC samples with PFS data in GSE102349, and found 11 protective and 17 risk ferroptosis regulators for PFS (Figure 7B). Interestingly, only ABCC1, TNFAIP3 and ALOX5 stood out when the differential expression and prognostic value were taken into consideration simultaneously. The highly heterogeneous expression of ferroptosis regulators between NPC and normal samples indicated that ferroptosis regulators might be of considerable importance in the occurrence and progression of NPC. Correlation analysis between ferroptosis regulators and immune cell infiltration levels was conducted to identify the candidate ferroptosis regulators associated with immune response (Figure 7C and Supplementary Table S6). We found that 14 DOFs (ZEB1, SAT1, NCOA4, MAPK3, IFNG, HMOX1, DPP4, CDO1, ATM, ATG7, ALOX5, ALOX15B, ALOX15, ACSL4) and 11 SOFs (ZFP36, TMBIM4, SLC7A11, SLC40A1, RB1, NQO1, MUC1, HIF1A, GCH1, CHMP5, ARNTL) were highly positively associated with immune cell infiltration, and 14 DOFs (VDAC2, TP53, RPL8, PEBP1, MYB, LONP1, KEAP1, IDH1, ELAVL1, EGFR, CS, ATP5G3, ACVR1B, ABCC1) and 11 SOFs (PROM2, PRDX6, OTUB1, NFS1, LAMP2, HSF1, GPX4, CISD2, CBS, CA9, ATF4) were highly negatively associated with immune cell infiltration. The same result could be found in correlation analysis between FEP score and above immune related ferroptosis genes (Figure 7D). To further identify feature ferroptosis genes, LASSO algorithm was performed and found seven ferroptosis regulators between two FEP score groups (Figures 8A,B). The seven ferroptosis genes signature, containing CDO1, TP63, STAT3, ELAVL1, CS, CISD2, ABCC1, showed a highest accuracy of 0.983 by the GFMM classifier in one of the 127 formulas, as shown in Figure 8C. The coefficients of genes involved in the signature was shown in Figure 8D, and the formula score was named simplified FEP (sFEP) score: sFEP score = CDO1 × 6.802 + TP63 × −1.681 + STAT3 × −1.752 + ELAVL1 × −3.525 + CS × −3.83 + CISD2 × −1.276 + ABCC1 ×−1.445. Interestingly, sFEP score showed slightly better function than FEP in prediction of PFS in NPC (Figure 8E), and we could find that the number of patients with disease progression decreased as sFEP score increased (Figure 8F). We developed a nomogram based on the Cox regression model to predict the 1- and 3-years PFS probability for NPC patients (Figure 8G). The calibration plots for the 1- and 3-years PFS showed an optimal agreement between the nomogram-predicted and observed PFS, which was used to evaluate the accuracy of the prediction signature (Figure 8H).

### Regulatory network between ferroptosis and m6A modification genes

**FIGURE 9:**
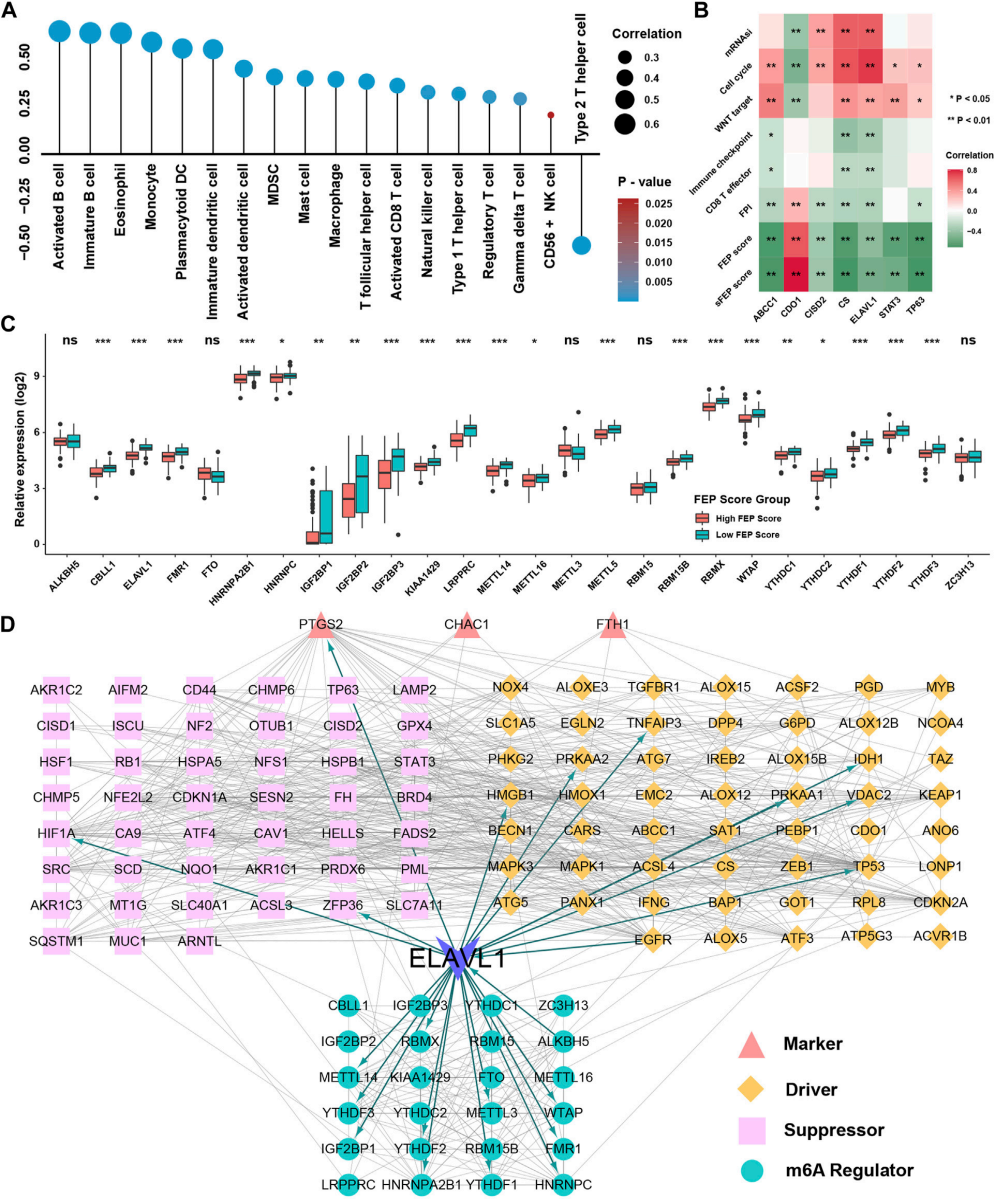
Construction of network among m6A regulators and ferroptosis regulators. **(A)** Correlation analysis between sFEP score and the immune cells infiltration levels. **(B)** Correlation analysis between seven ferroptosis genes and mRNAsi, FPI, FEP score and pathways. **(C)** The relative expression of m6A regulators in different FEP score groups. **(D)** Protein-protein interaction network among m6A regulators and ferroptosis regulators. The asterisks represented *p* values (*p < 0.05; **p < 0.01; ***p < 0.001) and ns represented no significance.

Given the similar ability of sFEP score with FEP score in terms of prognostic value, correlation analysis was performed and the result showed that sFEP score was also positively correlated with immune cell infiltration levels in NPC (Figure 9A). To further validate the biological function of these candidate ferroptosis related genes, correlation analysis showed that six signature genes (ABCC1, CISD2, CS, ELAVL1, STAT3, TP63) were positively correlated with mRNAsi, cell cycle and WNT signaling pathways and three genes (ABCC1, CS, ELAVL1) were negatively correlated with immune checkpoint and CD8 T cell effector (Figure 9B). As ELAVL1 is also a famous m6A regulatory gene (Chen Y. et al., 2019) and m6A modification could interplay with immune system and influence the infiltration of immune cells (Chen Y. G. et al., 2019), we speculated whether m6A regulators could interact with ferroptosis regulators and further be the underlying decipher for differentially expression of ferroptosis regulators. The result showed that m6A regulators were differentially expressed in FEP score groups with most of m6A regulators being low expressed in high FEP score group (Figure 9C). Protein-protein interaction network was employed to depict the landscape of m6A regulators and ferroptosis regulators, and ELAVL1 had dual identities among the network, which was not only a m6A reader but also a validated ferroptosis driver (Figure 9D). Correlation analysis further validated a high association between the expression of m6A regulators and the expression of ferroptosis regulators (Supplementary Figure S3). Taken together, the study strongly indicated that ferroptosis related genes were significantly correlated with tumor immune infiltrations and might be regulated by m6A modification.

## Discussion

Increasing evidence demonstrated the crucial role of ferroptosis in antitumor immunity as well as cross talk with various immune cells including cytotoxic T cells and macrophages (Dai et al., 2020; Shen et al., 2021). Different from most studies focusing on limited cell types or ferroptosis regulators, the present study comprehensively recognized the overall infiltration characterizations of immune cells mediated by integrative roles of multiple ferroptosis regulators. The study firstly revealed three distinct ferroptosis regulatory subtypes in NPC with distinct TME cell infiltration characterizations. Subsequently, FEP score system was further identified, and the

Associations between FEP score and immune cell infiltration, EBV infection and cancer stemness index were analyzed. Subtype 1 and subtype 3, as well as high FEP score groups were characterized by immune activation, corresponding to immune activated phenotype (Gajewski et al., 2013; Turley et al., 2015; Chen and Mellman, 2017), while subtype 2 and low FEP score groups were characterized by immune suppression, corresponding to immune suppressed phenotype (Kim and Chen, 2016). FEP score significantly positively correlated with immune checkpoint, CD8 T cell effector and antigen processing machinery, and higher FEP score was highly correlated with better immune therapy response.

Although NPC is a malignant tumor with relatively good prognosis under standard treatment, distant metastasis remains the main cause of treatment failure and death (Hong X. et al., 2020). The FEP score could well predict the risk of metastasis and reflect the clinical stage and previously defined TME subtype (Zhang et al., 2017). It is true that TME subtype could also reflect the TME infiltration and predict PFS in NPC (Zhang et al., 2017), but the construction of TME subtype differed completely and the function of TME subtype was partially distinctive from that of the FEP score. The PCA algorithm used in the present study advantages in retaining the most characterization of ferroptosis regulators in NPC, and that was why FEP score displayed high association with most of ferroptosis regulators. In terms of function, FEP score could well reflect the mRNAsi, TME infiltration and EBV genes. FEP score was negatively correlated with mRNAsi, an index to assess the stemness of cancer cells, which could explain why FEP score was a protective factor for metastasis, as cancer stem-like cells (CSCs) are supposed to participate in cancer metastasis and recurrence (Wei et al., 2014). Moreover, copy number alteration, including both deletion of inhibitors and amplification of activators in NF-κB signaling pathway could also be related with CSCs and poor prognosis. Immunotherapy has been suggested to contribute to developing more effective and safer treatment modalities in NPC in future (Hong et al., 2018; Chow et al., 2019; Masterson et al., 2020). According to our analysis, FEP score was apparently positively correlated with most of immune checkpoints such as TIM3, TIGIT, PD1, CTLA4 and LAG3, and the expression of ferroptosis regulators were highly associated with immune cell infiltrations. In combination with the results of TME infiltration, the prediction role of FEP score and ferroptosis regulators in efficacy of immunotherapy could be reasonable and obvious. EBV infection are assumed to activate the initiation of NPC through multiple pathways (Tsao et al., 2017). Although the expression of EBV genes could not be obtained, the EBV gene expression for NPC was robustly assessed based on GSVA analysis in GSE102349. EBV gene expression was also found to be correlated with “cold” TME infiltration negatively and h mRNAsi positively. However, the relationship between ferroptosis and EBV infection remained uncertain, which might be a novel research topic.

As NPC has some unique features, the role of ferroptosis in NPC also differs from other cancers. NPC was closely related with EBV, the first oncogenic virus identified in humans. Compared with those in other cancers, the role of EBV in tumorigenesis of NPC was quite clearer. Recent study has reported that EBV infection could reduce the sensitivity of NPC cells to ferroptosis by upregulating the expression of SLC7A11 and GPX4 expression, and high GPX4 expression was correlated with poor clinical outcomes, suggesting a novel target in the treatment of NPC (Yuan et al., 2022). This was consistent with our finding that EBV infection level was associated with ferroptosis levels and might be related with infiltration levels of immune cells. In addition, radiotherapy is the main effective treatment modality in NPC, which is different from many solid tumors that require surgery. The cross-link between ferroptosis and radio-sensitization of NPC are generally being studied, hoping that linking the mechanism of ferroptosis with radiotherapy strategies could accelerate the development of novel ferroptosis-based treatment in NPC (Li et al., 2021).

The biological function of ferroptosis varied among different types of tumors and could be seen in the field of drug resistance, immune evasion, antitumor effect or progression and metastasis. The differential expression analysis and survival analysis showed that the truly differentially expressed ferroptosis regulators with significant prognostic value were not abundant. Isocitrate dehydrogenase 1 and 2 (IDH1 and IDH2) are key catalytic enzymes that convert isocitrate to α-ketoglutarate, and small molecule inhibitors of mutant IDH1/2 enzymes represent a novel class of drug for targeted therapy for patients harboring IDH1/2 mutations (Mondesir et al., 2016). The well-known multidrug resistance-associated protein 1 (ABCC1) is a major player in cancer related multidrug resistance and has been well investigated in the management of drug-resistant tumors (Wiese and Stefan, 2019). TNFAIP3, an inflammation-related gene, could inhibit migration and invasion in NPC by suppressing epithelial-mesenchymal transition (EMT) (Huang et al., 2017), and EBV infection could decrease the expression of TNFAIP3 in NPC tumors (Xu et al., 2019). Moreover, arachidonate 5-lipoxygenases (ALOX5) could enhance the function of macrophages in the changing tumor environment (Weigert et al., 2018). Even though all these genes have been supposed to be associated with ferroptosis recently in other types of cancer, none of them have been investigated in NPC regarding ferroptosis or immune infiltration, needing further validation with basic experiments. Using machine learning algorithm LASSO, we also identified candidate ferroptosis related genes, which might be related with candidate ferroptosis regulators. Furthermore, the study made a novel attempt to investigate the relationship between m6A modification and ferroptosis regulators to uncover the underlying regulatory mechanisms of ferroptosis in NPC.

In conclusion, the FEP score could be used to comprehensively evaluate the ferroptosis regulatory patterns and their corresponding characterization of immune cell infiltration in TME within individual patient, and further to decide the immune phenotypes of tumors and predict patients’ response to immunotherapy to guide more effective clinical practice. This study has also provided new insight into cancer immunotherapy that targeting ferroptosis regulators or FEP phenotype-related genes to change the ferroptosis regulatory patterns and further reverse the adverse TME cell infiltration characterization, contributing to the development of novel immunotherapeutic agents or combination therapy.

## Data Availability

The datasets presented in this study can be found in online repositories. The names of the repository/repositories and accession number(s) can be found in the article/Supplementary Material.
