# Supplementary material for: Ferroptosis regulators related scoring system by Gaussian finite mixture model to predict prognosis and immunotherapy efficacy in nasopharyngeal carcinoma
Source: Front Genet. 2022 Sep 2;13:975190. doi: 10.3389/fgene.2022.975190 (PMC9479336; doi:10.3389/fgene.2022.975190)
Supplement: Supplementary file 2 [file Image1.pdf]

Supplementary materials

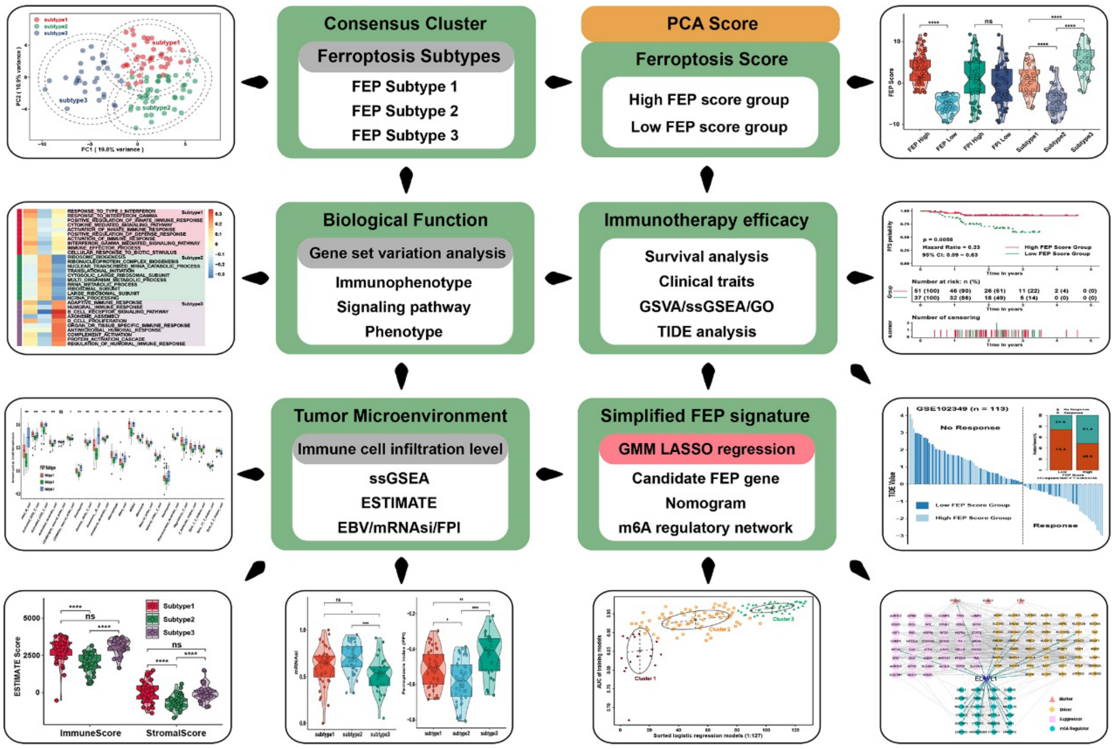

Figure S1. Flow chart of this study.

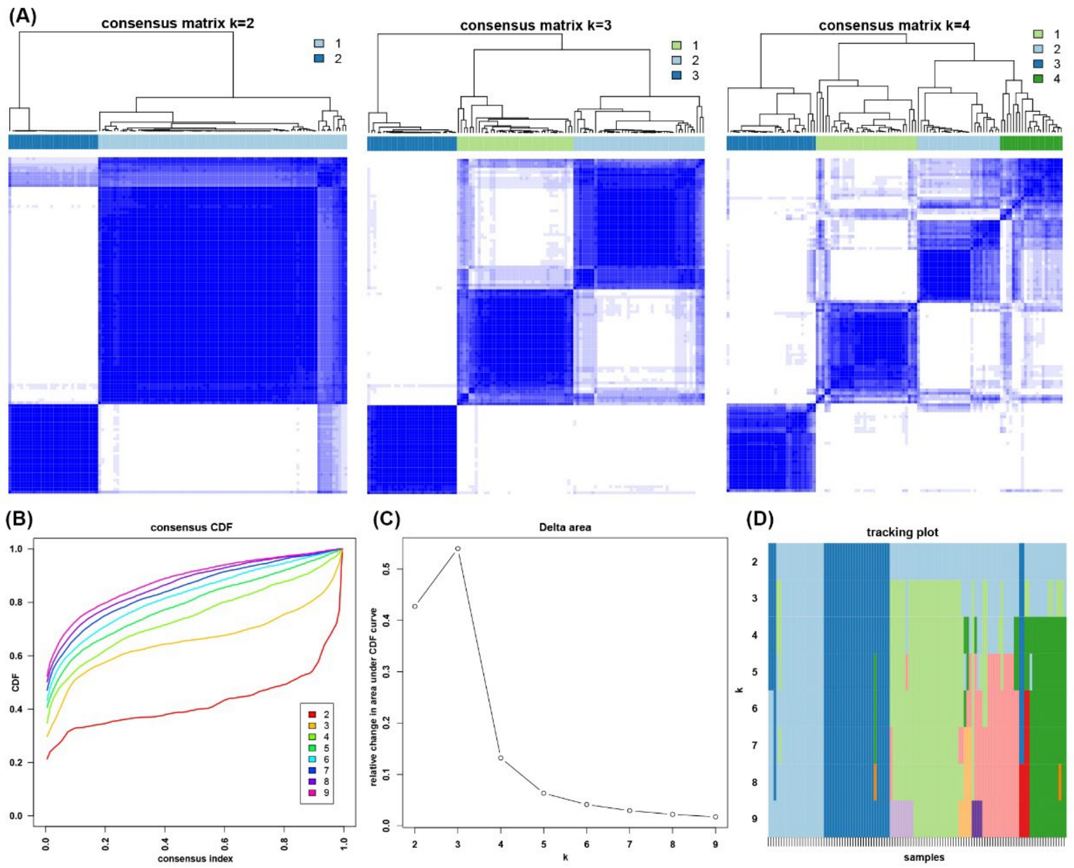

Figure S2. Unsupervised clustering of 113 ferroptosis regulators in GSE102349 cohort. A Consensus matrices of the GSE102349 cohort for k = 2 – 4. B-D Consensus cumulative distribution function (CDF) to look for the best k.

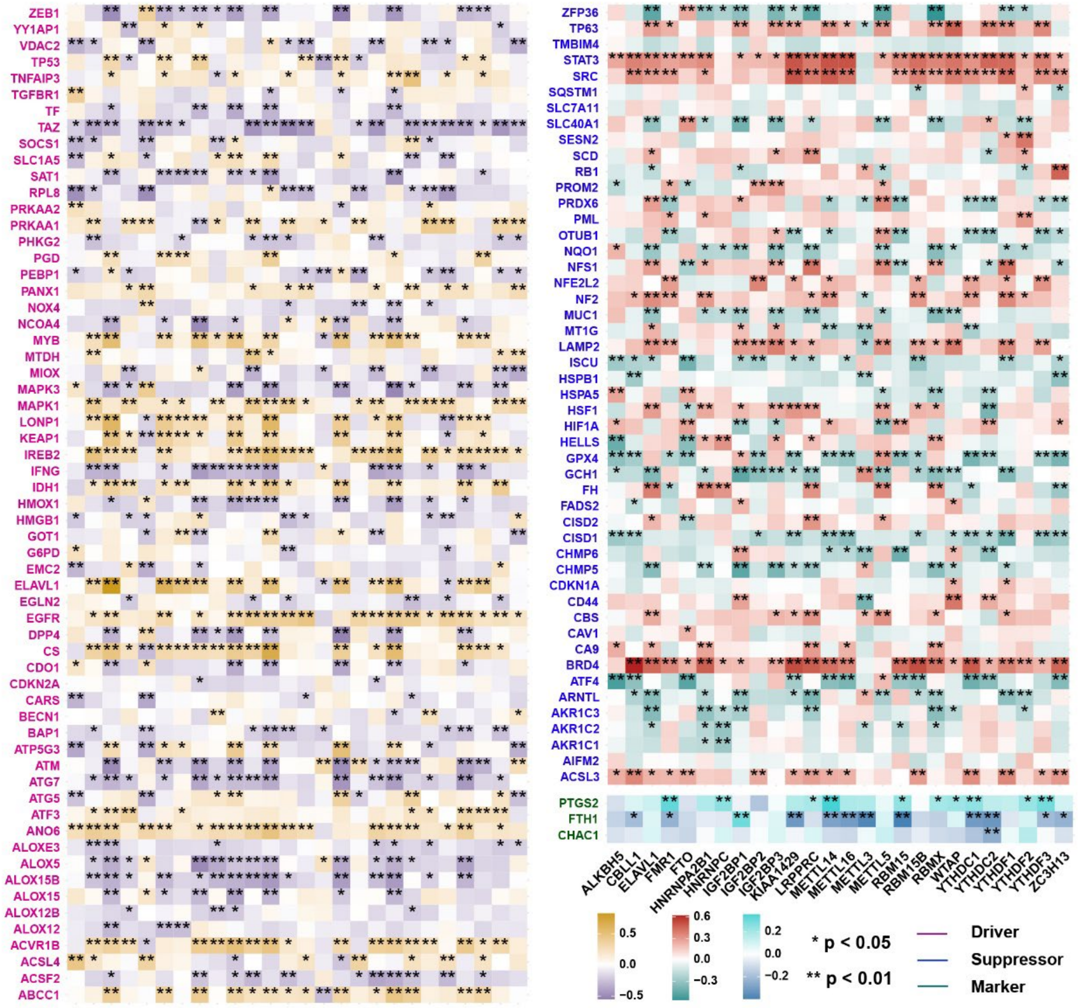

S3. Construction of network among m6A regulators and ferroptosis regulators. Correlations between ferroptosis regulators and m6A regulators in NPC using Spearman analysis. The asterisks represented P values (*P < 0.05; **P < 0.01).
